# Developmental delay in a resource-constrained environment: Screening, surveillance and diagnostic assessment

**DOI:** 10.4102/safp.v63i1.5306

**Published:** 2021-05-26

**Authors:** Vasantha Govender, Deshini Naidoo, Pragashnie Govender

**Affiliations:** 1 Department of Paediatric Neurology, KwaZulu-Natal Children’s Hospital, Durban, South Africa; 2 Department of Paediatric Neurology, Inkosi Albert Luthuli Hospital, Durban, South Africa; 3 Discipline of Occupational Therapy, School of Health Sciences, University of KwaZulu-Natal, Durban, South Africa

**Keywords:** developmental delay, paediatric screening, resource constrained, diagnostic assessment, early identification

## Abstract

The range and severity of developmental delays vary, and a systematic approach to ensuring early detection for early intervention is essential. The formative years are considered critical for nurturing and maximising developmental potential. In this article, the authors describe a clinical approach to developmental delay within resource-constrained environments of South Africa. The article unpacks the history and examination, developmental screening, surveillance and diagnostic assessment and social determinants of health. For timely interventions to occur, early and accurate assessment is necessary. Medical officers and other health professionals such as nurses, general practitioners and therapists working in low-resourced contexts may use this information in their approach to the assessment of developmental delay.

## Introduction

There have been tremendous efforts in the last decade in child health in meeting the goals of early identification and intervention for children with developmental disorders.^1^ Support and early detection ensure that these children have the opportunity to thrive in a very critical period of development. Developmental delays (DDs) range in severity with several underlying aetiologies. These may include prenatal factors such as genetics (including metabolic), malformations and toxins; perinatal factors such as hypoxia and prematurity; and postnatal (acquired) factors such as infections and status epilepticus.^2^ Nutrition, growth and other factors have also been associated with DD.^3,4^ Outcomes in development should not be viewed in isolation, but with the interactions of the various risks and protective factors that influence early child development.^5^

## Developmental assessment

The developmental assessment for children presenting with DD can be approached from a perspective of screening of development and social determinants of health (SDOH), developmental surveillance and more comprehensive assessments including diagnostic assessments. There are several variations in normality and deviations from typical development, which need to be considered before labelling a child as delayed.^6^ These delays become significant when less than two standard deviations or 50% less than expected are achieved and global delays if two or more domains are affected.^6^ The domains of functioning include activities of daily living, social interaction, gross and fine motor skills, language and communication and cognition and behaviour.^6^

## Developmental screening and surveillance

Current evidence indicates that developmental screening and surveillance are problematic in rural primary healthcare (PHC) facilities in South Africa.^7^ Developmental surveillance should occur routinely at specific vaccination dates (9 and 18 months) and ideally at two years and three years for early detection/identification of possible DD and before starting school at five years.^1^ Clinicians should closely monitor children and families with risk factors such as prematurity, low socioeconomic factors, teenage pregnancy and poor education levels of parents.^8^ Information should be collated from multiple sources, including the parents, caregivers and teachers/early childhood development practitioners.^6^ During surveillance and developmental screening, a direct referral may be necessary for a more comprehensive assessment by the team members. This may be a referral to a paediatrician, occupational therapist, physiotherapist, speech-language therapist, audiologist and/or psychologist.

A paediatric human immunodeficiency virus (HIV) care developmental assessment toolkit has been developed for the South African context.^9^ Although this tool was designed for children with HIV, the toolkit covers the domains of functioning and potential red flags that can be considered for children without HIV. Developmental screening using parental reports and other screening instruments can be beneficial and provide relevant developmental cues.^8^ The Parents Evaluation of Developmental Status (PEDS),^10^ Ages and Stages questionnaire (ASQ)^11^ and the Ten questions (TQs)^12^ are parental/caregiver screening tools that have been used within the South African setting, but the former two are at a cost and may not be feasible in low- and middle-income countries (LMICs). Feasibility of developmental screening tools used in LMICs at a PHC has been previously reported.^13^ Additional screening tools for LMICs such as the Red Cross War Memorial Children Hospital Developmental Screening Tool for Neurodevelopmental Delay in HIV-Infected South African Children^14^ and Guide for Monitoring Child Development^15^ are free and do not require formal training. The Modified Checklist for Autism in Toddlers-Revised can be used for screening for autism spectrum disorder (ASD).^16^ Screeners for SDOH for children have been comprehensively described in a systematic review by Sokol and colleagues.^17^ These may be useful when considering children in the low-resourced environment.

## History and examination

**FIGURE 1:**
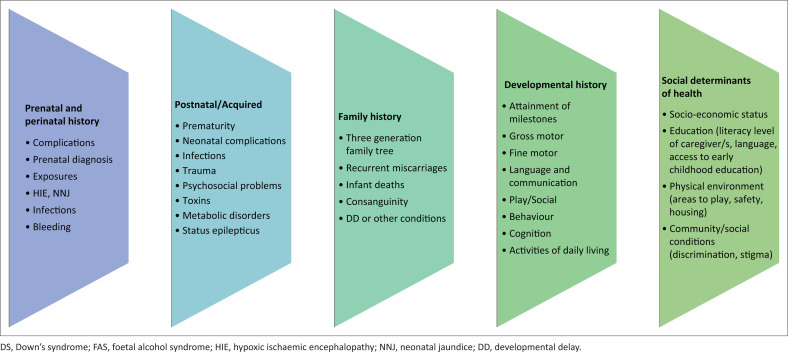
Aspects to consider when obtaining a developmental history.

A comprehensive history about DD should be elicited as a starting point for assessment (Figure 1).^17,18,19^ Taking a detailed chronological history of all developmental milestones is essential to identify the delay. The history and examination alone, if done correctly, can help identify up to 40% of the aetiology of DD.^19^

In addition, one should actively take a history of seizures as this is an important cause of DD and regression. This includes an inquiry of subtle seizures such as epileptic spasms, atonic seizures or drop attacks and staring (absences). An important part of the history is to search for red flags, which are warning signs if certain milestones are not achieved, for example no head control by 4 months, not sitting by 9 months, not babbling by 1 year or no single words by 16 months.^8^ Developmental regression is an immediate referral criterion for diagnostic workup as it can signify a serious metabolic or neurodegenerative problem or epilepsy. Suspect inborn errors of metabolism (IEM) if there is a history of neonatal deaths, consanguinity, failure to thrive, recurrent vomiting, episodic fluctuations in lethargy and coma.^20^ Suspect ASD on history if there is speech delay, temper tantrums on changing routine, repetitive, restricted behaviour and isolated play.^21^

## Diagnostic assessments

**FIGURE 2:**
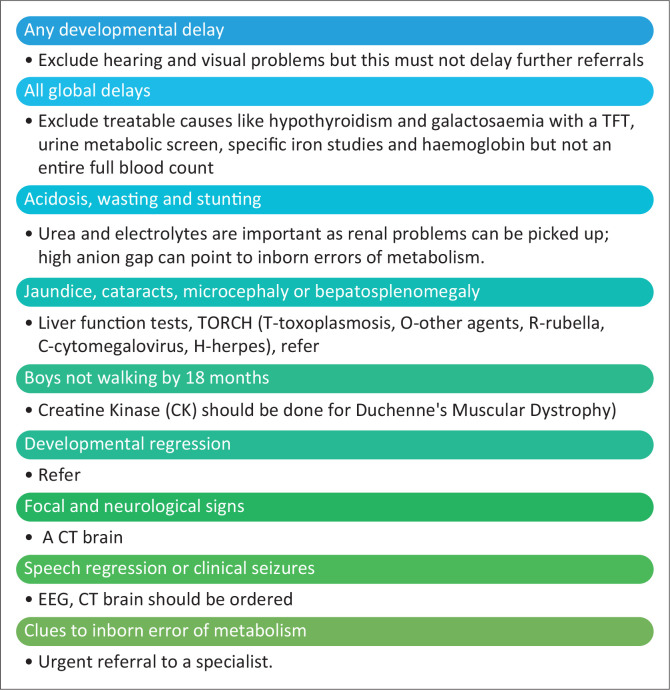
Considerations in the diagnostic assessment. TFT, thyroid function test; CT, computed tomography; EEG, electroencephalogram.

Plotting the weight and height is important as stunting and malnutrition signify a chronic disease. Look for pallor as it can be a sign of iron deficiency or malignancy. Examination for abnormalities of head size, asymmetry of the body, neurocutaneous lesions, impaired vision and poor hearing is vitally important, as it will guide one to further investigations. Early closure of sutures before 18 months can be a sign of craniosynostosis, which needs an early referral for investigation. Do a head-to-toe examination for dysmorphology. Any abnormal neurological sign like hypotonia with decreased reflexes can indicate the underlying aetiology like muscular disorders whilst hypertonia and brisk reflexes can point to brain damage such as cerebral palsy and neurodegenerative disorders. The approach to a diagnostic assessment and investigation is shown in Figure 2.

There are several algorithms and detailed evaluation plans of investigations in developed countries,^22,23^ but resources have to be considered in the approach taken for a low-resourced environment (Figure 2). A genetic testing protocol has been developed in KwaZulu-Natal, South Africa (Yates and Naicker, 2021, personal communication), which specifies that when a child is dysmorphic do not do a chromosome analysis or karyotype unless there is ambiguous genitalia or signs of Down’s syndrome or other trisomies, and DNA polymerase chain reaction (PCR) should be performed first (Table 1). The dysmorphic child should be evaluated by a paediatrician and referred to a genetic specialist or neurologist if no syndrome is identified. Although more expensive than chromosome analysis, Chromosomal microarray, has a 40% – 50% diagnostic rate and a 6-month turnaround time as compared with karyotyping, which has a 3% yield and 3-month turnaround time (Yates and Naicker, 2021, personal communication).

**TABLE 1:** Order of genetic testing.

Suspected diagnosis (on clinical features)	Genetic test	Next action
Down’s syndrome (T21)	QF-PCR (EDTA via SANBS) If ABNORMAL, send LiHep for karyotype	If QF-PCR normal → review patient (pt) and assess whether referral to genetics vs watch and wait DO NOT DO CHROMOSOMES
Edward syndrome (T18)	QF-PCR (EDTA via SANBS) If ABNORMAL, send LiHep for karyotype	If QF-PCR normal → review patient and assess whether referral to genetics vs watch and wait DO NOT DO CHROMOSOMES
Patau syndrome (T13)	QF-PCR (EDTA via SANBS) If ABNORMAL, send LiHep for karyotype	If QF-PCR normal → review patient and assess whether referral to genetics vs watch and wait DO NOT DO CHROMOSOMES
Turner syndrome	QF-PCR (EDTA via SANBS) If ab ABNORMAL N, send LiHep for karyotype	If QF-PCR normal → review patient and assess whether referral to genetics vs watch and wait DO NOT DO CHROMOSOMES
DSD/Ambiguous Genitalia	Urgent karyotype and request analysis for SRY	Refer to paediatric endocrinology
Developmental Delay + Dysmorphic syndrome not recognised known/suspected syndrome for which testing via NHLS is *not* available	None DO NOT DO QF-PCR (EDTA via SANBS)	Refer to genetics team at IALCH (ideally via DoH VULA App with photos and clinical details)
Subtle dysmorphic features in neonate/child who is neurologically normal and otherwise healthy	-	Review in neonatal/paediatric clinic Refer to genetics if concerns persist

*Source:* Adapted from Yates and Naicker (personal communication, 2021)

Note: Reproduced with permission from Yates and Naicker (personal communication, 2021).

SANBS, South African National Blood Services; QF-PCR, quantitative fluorescence-polymerase chain reaction; EDTA, ethylenediamine tetraacetic acid; DSD, differences in sex-development; SRY, sex-determining region Y gene; NHLS, National Health Laboratory Service; IALCH, Inkosi Albert Luthuli Central Hospital; DoH, Department of Health.

Fragile X syndrome has non-specific signs, so advocating routine screening in all children with an intellectual disability is not feasible as the pickup rate is 1.4% for boys and 0.9% for girls.^24^ The yield for metabolic testing is low (1% – 5%) and variable,^22^ but there is an endeavour to identify treatable intellectual disorders (www.treatable-ID.org) as long-term prognosis can be improved.^20^ Many of the disorders are rare, but investigations can be guided by clinical features such as early-onset seizures, encephalopathy, ataxia, skin rash, eye signs, hearing loss, coarse facial features, movement disorders such as dystonia, hypotonia, visceromegaly, progressive intellectual and neurological deterioration, late-onset behaviour changes, prominent expressive language delay and dysmorphism. Urgent referrals in these cases are essential.

## Conclusion

Constant surveillance and appropriate screening are essential to detect children with developmental problems that need further evaluation. History taking and comprehensive examination are essential. A clinical approach to diagnostic assessments and investigation is what is required in a resource-constrained environment. Ethical issues arise if one screens a child and does not do comprehensive assessments, designs suitable interventions and institutes timely and appropriate care.^18^ Comprehensive tracking and follow-up systems are needed to ensure that those children identified through surveillance, screening and assessments receive appropriate services at all levels of care.^25^
